# Pulmonary tumour embolism and lymphangitis carcinomatosa: a case report and review of the literature

**DOI:** 10.1186/s13019-022-01832-8

**Published:** 2022-05-07

**Authors:** Jan Engel, Johann Auer

**Affiliations:** Department for Cardiology and Intensive Care Medicine, St Josef Hospital, Ringstraße 60, 5280 Braunau, Austria

**Keywords:** Pulmonary tumour embolism, Lymphangitis carcinomatosa, Acute right heart failure

## Abstract

**Background:**

Pulmonary tumour embolism and lymphangitis carcinomatosa are complications of malignancy that may mimic the clinical presentation of pulmonary embolism.

**Case presentation:**

We present the case of a 52-year-old male patient with acute-onset right ventricular strain and dyspnoea with elevated D-dimer and without signs of pulmonary embolism on computed tomography pulmonary angiogram (CTPA) and ventilation/perfusion scintigraphy. The patient died eleven days after initial presentation. The diagnosis of pulmonary tumour embolism and lymphangitis carcinomatosa due to carcinoma of unknown origin was made post-mortem by immunohistochemical examination.

**Conclusion:**

Pulmonary tumour embolism and lymphangitis carcinomaosa are complications of malignancy and potential causes of acute right ventricular strain. Radiological signs are unspecific and the clinical course usually fatal. These differential diagnoses should be considered in patients with acute right ventricular strain, dyspnoea and positive D-dimer if there are no signs of pulmonary embolism on CTPA.

## Introduction

Right ventricular strain with acute onset dyspnoea is a hallmark of various conditions, including acute pulmonary embolism (PAE), myocarditis, acute respiratory distress syndrome (ARDS) and acute-onset cardiac shunts. Medical history, blood tests and imaging are usually characteristic for each condition and enable focused management of patients. Here we present a rare cause of acute onset right heart failure.

## Case report

### History

We present a 52-year-old man with no prior medical conditions and no regular prescription or over the counter medication. He initially presented to our general surgery department with diffuse abdominal pain, obstipation and a history of weight loss of 8 kg over eight months.

Three weeks before admission, the patient had consulted a pulmonologist because of a chronic cough. A chest X-ray and spirometry had been unremarkable, and a short course of inhaler therapy with salmeterol and fluticasone propionate resulted in complete resolution of symptoms.

On admission, blood tests showed a raised CRP (96.1 mg/l), a mild leucocytosis (13.85 × 10^3^/mcl) and normochromic normocytic anaemia (Hb 13.7 g/dl). Empiric antibiotic therapy was started with cefuroxime and metronidazole. Coloscopy was performed, which was unremarkable. A computed tomography (CT) of the abdomen was performed, which showed pathologically enlarged retroperitoneal lymph nodes and ascites.

### Presentation

**Fig. 1:**
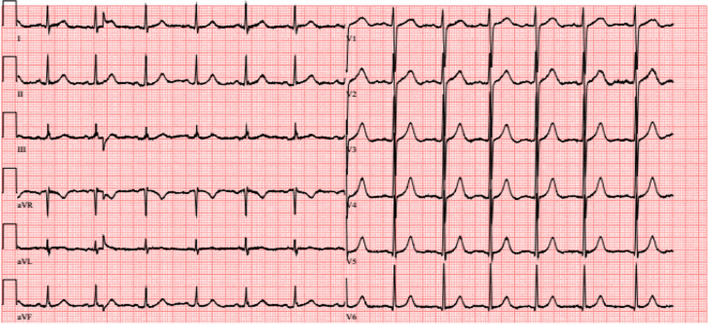
ECG on admission, unremarkable sinus rhythm

**Fig. 2:**
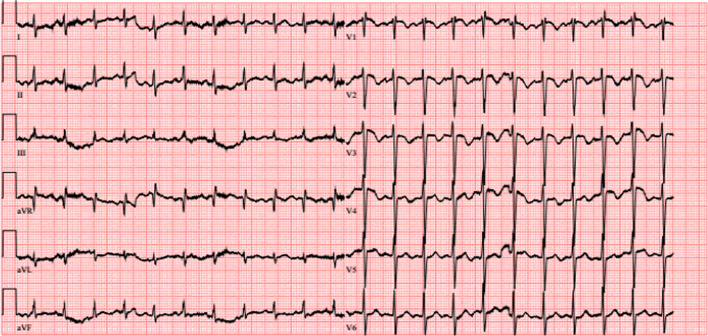
ECG after onset of sudden dyspnoae showing negative T-waves in V1–V3

The patient was transferred to our oncological department on suspicion of lymphoma for further evaluation of the findings when he developed sudden-onset dyspnoea with a respiratory alkalosis (pH 7.48, pO2 56 mmHg, pCO2 25 mmHg, BE -3,3). An electrocardiogram (ECG) showed new onset negative T-waves in V1–V3 (Figs. 1, 2).

**Fig. 3:**
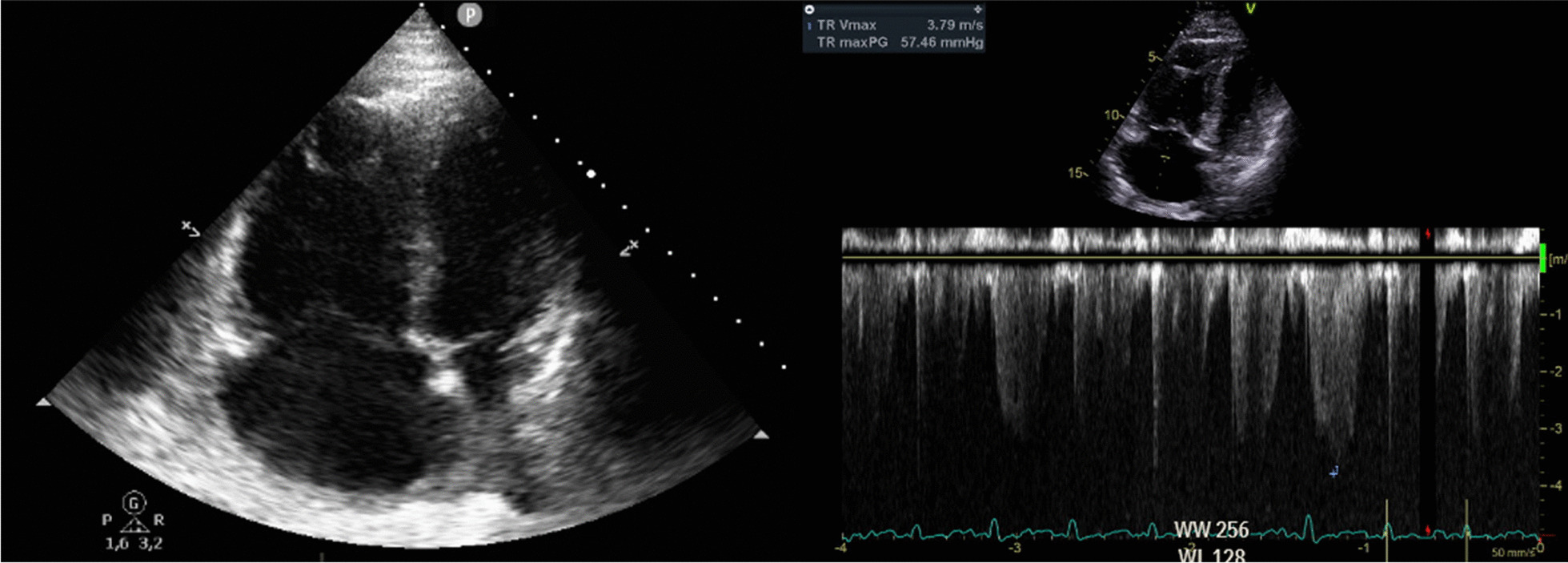
Echocardiography showing acute right heart dilatation

Echocardiography revealed a massively dilated right ventricle with reduced right ventricular function (TAPSE 1.5 cm) and an estimated systolic pulmonary artery pressure of 70 mmHg (Fig. 3).

**Fig. 4:**
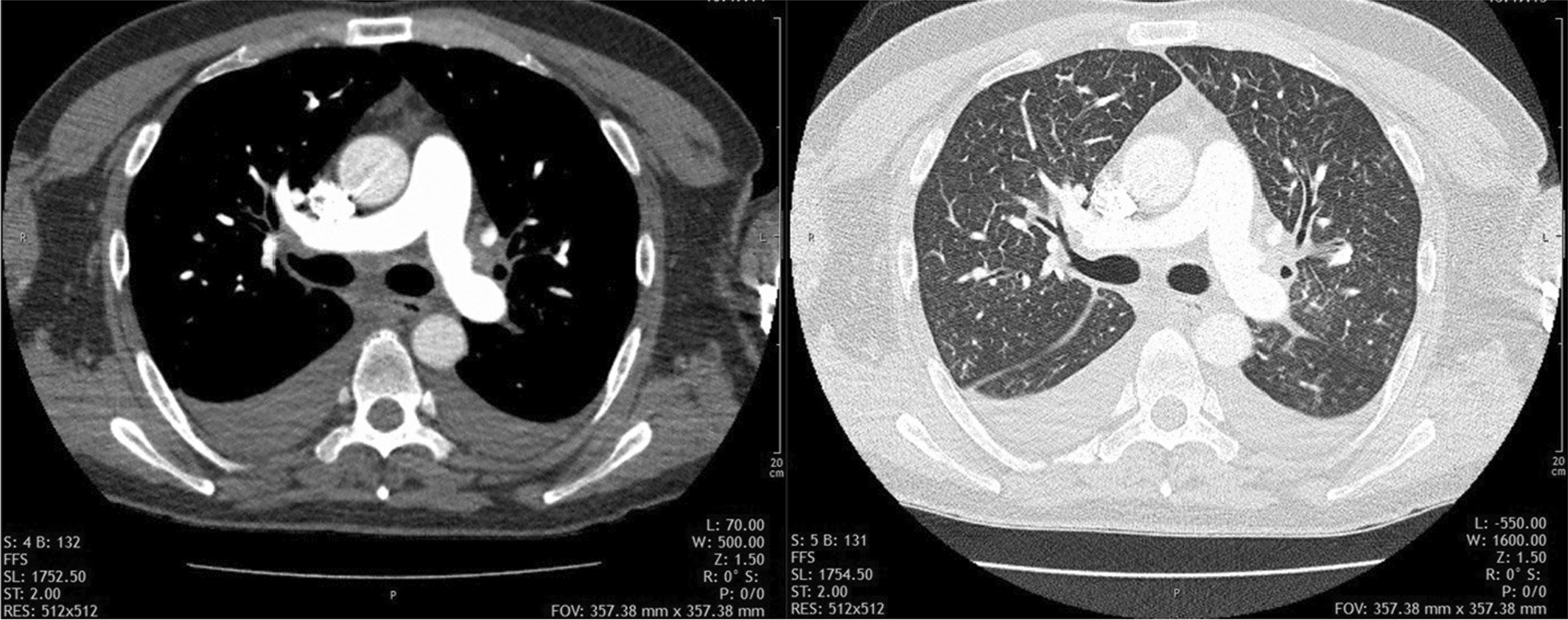
CTPA showing no signs of PAE, diffuse, small ground-glass opacifications, and bilateral pleural effusions

A computed tomography pulmonary angiogram (CTPA) was performed, which showed no signs of PAE or deep vein thrombosis (DVT), but small areas of ground-glass densities throughout both lungs and bilateral pleural effusions (Fig. 4).

Laboratory tests showed a substantially increased NT-pro-BNP (17,126 pg/ml) when compared to the base value on admission (557.8 pg/ml) and a positive D-dimer (11.6 mg/l). Serum high-sensitivity troponin I (hsTnI) was modestly raised (123.2 pg/ml) but remained stable in subsequent controls.

### Progress

**Fig. 5:**
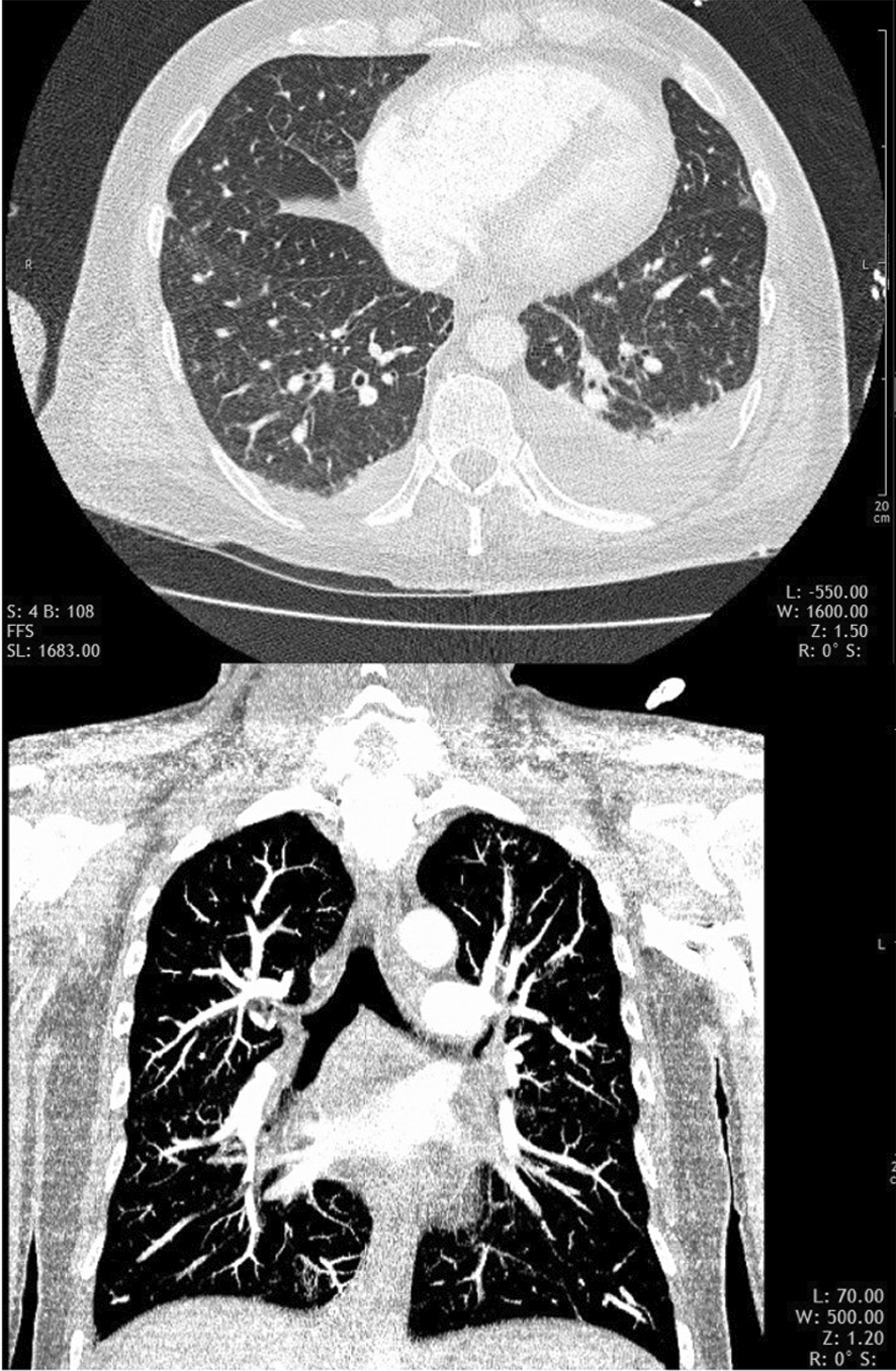
Follow-up CTPA showing pulmonary findings

Subsequently, the patient's oxygen demand increased to 8 l/min, and echocardiography continued to show right ventricular strain. Due to the patient's presentation and the sudden onset of symptoms, but again there were no signs of pulmonary artery obstruction. Moreover, there was no progression of the other pulmonary findings (Fig. 5).

**Fig. 6:**
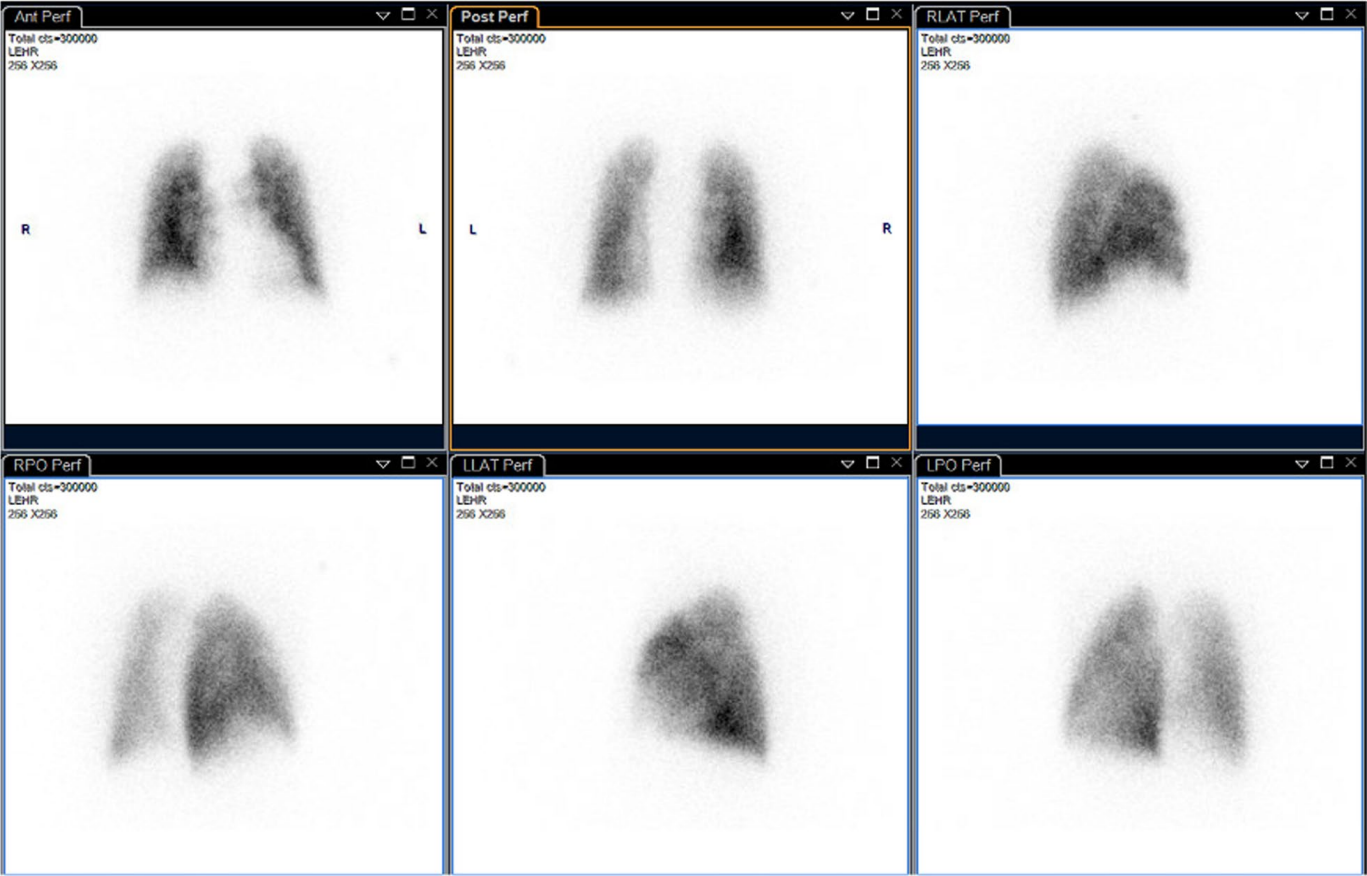
scintigraphy showing non-specific, diffuse perfusion defects

Perfusion/ventilation scintigraphy was performed, which showed diffuse subtle perfusion defects throughout both lungs, compatible with mild bronchial obstruction rather than PAE (Fig. 6).

A diagnostic thoracocentesis to determine the cause of the pleural effusion showed a haemorrhagic exudate with 10,000/3 erythrocytes, 1819/3 leukocytes and 80% uncharacteristic mononuclear cells. A gram stain was negative.

The differential blood count and immunofixation showed no relevant or specific findings (total protein 64 g/l, IgA < 7 mg/dl, IgG 1560 mg/dl, IgM 104 mg/dl, haptoglobin 314 mg/dl, B2-MG 2530 mcg/l). ANA-and ANCA-screening was negative. T-PSA (1.88 ng/ml), CEA (3.3 ng/ml) and CA 19-9 (8.8 U/ml) were within the normal range. CRP was falling under empiric antibiotic therapy (CRP 17.6 mg/l).

The patient remained dependent on supplemental oxygen.

BNP and hsTnI showed a downward trend on the third day since symptom onset (NT-proBNP 557.8 pg/ml; hsTnI 20.4 mg/dl).

Subsequently, the patient developed flank pain, and sonography showed hydronephrosis due to obstruction of the ureter by the massive retroperitoneal lymphadenopathy. The patient had to be transferred to an external urology department. Shortly after the transfer, the patient suffered an asystolic cardiac arrest and died despite resuscitation efforts and.

### Post-mortem

**Fig. 7:**
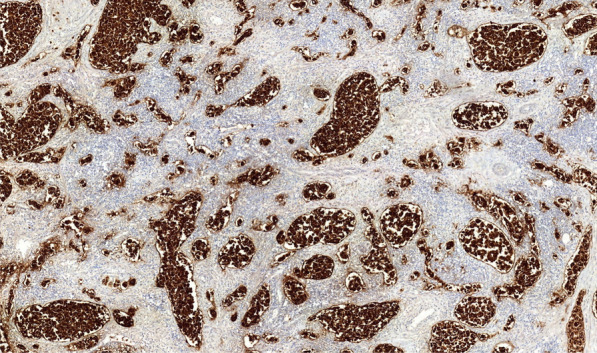
EPCAM stain showing carcinoma cells obstructing the pulmonary micro vasculature

**Fig. 8:**
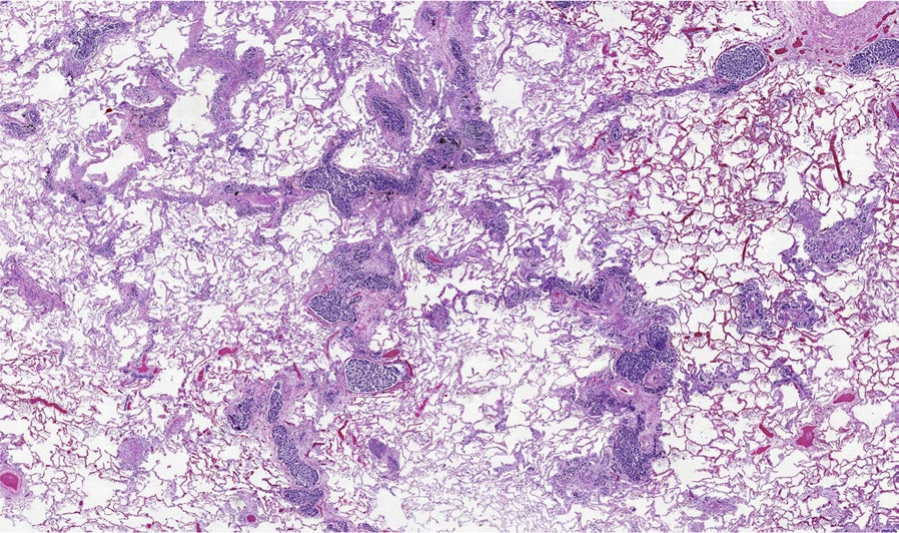
HE, lymphangitic pulmonary infiltration

**Fig. 9:**
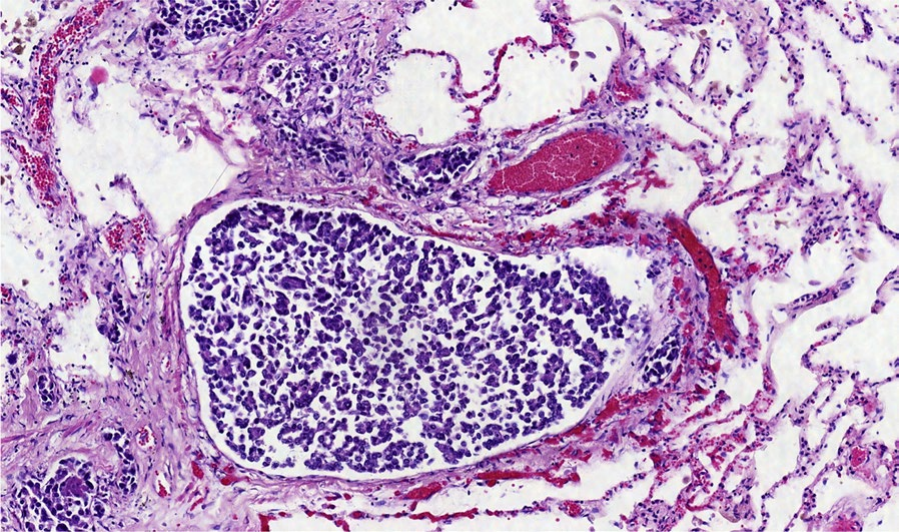
HE, micro thrombus within pulmonary vasculature, lymphangitic pulmonary infiltration

The autopsy showed massive retroperitoneal lymphadenopathy due to disseminated infiltration with poorly differentiated lymphoreticular carcinoma tissue, concordant with infiltrative lymphangitis. In correlation with this finding, the lungs showed substantial angio-invasive tumour infiltration with poorly differentiated carcinoma tissue of the peripheral microvasculature (Figs. 7, 8, 9). Notably, no embolic material was found in any proximal pulmonary arteries. An immunomorphological examination could not identify a primary tumour. There was no other organ involvement. Specifically, the pancreas, the gastrointestinal system, the biliary system, the prostate and the bladder were free from malignancy.

In conclusion, the pathological diagnosis was a carcinoma with unknown primary (CUP), with angio-invasion of carcinoma cells into the lung and the retroperitoneal lymph nodes, corresponding with tumour micro embolization and lymphangitis carcinomatosa.

## Discussion and review

Malignancy is a well-established risk factor for thrombotic complications, namely PAE. It can, however, also cause pulmonary hypertension and right heart strain by intravascular dissemination of tumour cells. Angio-invasive spreading of tumour cells in the form of pulmonary tumour micro embolization and lymphangitis carcinomatosa are established complications of malignancy where metastatic cells are disseminated into pulmonary blood and lymphatic vessels, respectively, distinguished from pulmonary metastasis by the absence of tumour infiltration of lung parenchyma and formation of a solid mass. Both entities are similar in manifestation, prognosis and management and merely represent ends on a spectrum of possible histological manifestations. Overlap syndromes regularly occur, as was the case in this patient, and terminology is often used interchangeably in the literature. Lymphangitis carcinomatosa usually presents with slowly progressive dyspnoea over months to years, whereas pulmonary tumour micro embolism (PTE) and angio-invasive tumour spread can present with right ventricular strain [1].

Imaging is only of limited value in the diagnosis of PTE and lymphangitis carcinomatosa. Unspecific signs like hilar lymphadenopathy, pleural effusions, ground-glass opacities, and tree-in-bud configurations have been described in the literature [2–4].

Perfusion/ventilation scintigraphy typically shows only minor, diffuse perfusion defects throughout both lungs [5].

Specific diagnosis of tumour pulmonary micro embolism can be achieved either by transbronchial lung biopsy or aspiration of blood from the pulmonary arteries using a wedged right heart catheter [6].

There are few therapeutic interventions beyond hemodynamical and respiratory support. Early chemotherapy, pulmonary vasodilators and glucocorticoids are used to treat PTE. However, the clinical course is usually very poor despite therapeutic efforts [6].

A recent review of 107 case reports of lymphangitis carcinomatosa between 1970 and 2018 showed that it is caused mainly by malignancies of the breast, stomach and lung. It was shown that the average time of survival was 158 days after the onset of mild early symptoms like dyspnoea and dry cough [7]. In patients with PTE, an autopsy series in twenty patients between 1951 and 1990 showed a mean time of survival of one month after onset of respiratory symptoms [8].

## Conclusion

PTE and lymphangitis carcinomatosa are rare causes of respiratory insufficiency and pulmonary hypertension and usually associated with a gradual onset of symptoms. This case report shows that the diagnosis should also be considered in patients with suspected or confirmed malignancy with acute onset right heart failure and negative imaging for PAE.

## Data Availability

Radiological and histological images were provided by the Radiology department of the St Josef Hospital Braunau and the Pathology department of the Barmherzige Schwestern hospital Ried.
